# Whole-Genome Sequencing of Streptomycin-Resistant Mycobacterium tuberculosis Strain SBH145 from Sabah, Malaysia

**DOI:** 10.1128/mra.01040-21

**Published:** 2022-01-06

**Authors:** Zainal Arifin Mustapha, Jaeyres Jani, Cheronie Shely Stanis, Dg Syahidah Nadiah Abdull Majid, Chin Kai Ling, Roddy Teo, Kamruddin Ahmed

**Affiliations:** a Department of Medical Education, Faculty of Medicine and Health Sciences, Universiti Malaysia Sabah, Kota Kinabalu, Sabah, Malaysia; b Borneo Medical and Health Research Centre, Faculty of Medicine and Health Sciences, Universiti Malaysia Sabah, Kota Kinabalu, Sabah, Malaysia; c Department of Biomedical Sciences, Faculty of Medicine and Health Sciences, Universiti Malaysia Sabah, Kota Kinabalu, Sabah, Malaysia; d Tuberculosis and Leprosy Control Unit, Sabah State Health Department, Ministry of Health Malaysia, Kota Kinabalu, Sabah, Malaysia; e Department of Pathology and Microbiology, Faculty of Medicine and Health Sciences, Universiti Malaysia Sabah, Kota Kinabalu, Sabah, Malaysia; University of Maryland School of Medicine

## Abstract

This paper reports on the whole-genome sequencing of a streptomycin-resistant Mycobacterium tuberculosis strain that was isolated from a patient with pulmonary tuberculosis in Sabah state of Malaysian Borneo. The strain belongs to the EAI2-Manila family of lineage 1 and is clustered with M. tuberculosis strains from the Philippines, India, and Taiwan.

## ANNOUNCEMENT

The burden of tuberculosis is high in Sabah state of Malaysia (1). To date, streptomycin-resistant Mycobacterium tuberculosis strains have not been reported. Here, we report for the first time the whole-genome sequence analysis of a streptomycin-resistant M. tuberculosis strain from Sabah. Streptomycin is not included in standard treatment regimens but is used in retreatment and as a first-line agent against drug-resistant tuberculosis (2).

Tuberculosis was diagnosed in an 81-year-old female patient from Kota Kinabalu, Sabah, Malaysia, with the GeneXpert MTB/RIF test. The strain was grown in a Bactec MGIT 320 system. Using a Bactec MGIT 960 SIRE kit with streptomycin, isoniazid, rifampin, and ethambutol, the strain was found to be streptomycin resistant. The genomic DNA was extracted using a Masterpure complete DNA and RNA purification kit. The quality was determined with a NanoDrop 2000c spectrophotometer.

DNA libraries were prepared using a NEBNext Ultra kit and sequenced on an Illumina HiSeq 4000 system. The sequencing output was 9,773,850 paired-end 150-bp reads, with a genome coverage of 415×. The quality of the sequence reads was checked using FastQC (https://www.bioinformatics.babraham.ac.uk/projects/fastqc), and the reads were preprocessed using BBMap v38.43 with a Phred score of Q30. SPAdes v3.11.1 (3) was used for *de novo* assembly, which generated 125 contigs, with an *N*_50_ value of 97,881 bp. The 99% draft genome size was 4,401,196 bp, with a GC content of 65.57%. NCBI Prokaryotic Genome Annotation Pipeline (PGAP) (4) was utilized to annotate the generated contigs.

For variant-calling analysis, the raw sequence reads were aligned to a reference genome (M. tuberculosis H37Rv, GenBank accession number NC_000962.3) by BWA MEM v0.7.1231 (5), in SAM-BAM format. This format was converted into readable sequences and alignment was done using SAMtools v0.1.1932 (6). Next, the Genome Analysis Toolkit v3.4.033 (7) was used for local realignment of the sequence reads. Finally, an average mapping rate of more than 99% with respect to the reference genome was generated for the reports in variant-calling analysis. The following criteria were used to filter variant sites: the quality of alignment had to be >50 bp or the quality of the base had to be >20 bp with >10 reads covering each site. For the annotation of single-nucleotide polymorphisms (SNPs), SnpEff v4.134 (8) was used. Moreover, the variant-calling analysis identified a mutation site in the *rpsL* gene (AAG→AGG, locus K43R), which confers high-level resistance to streptomycin (9). Streptomycin interferes with 16S rRNA and interferes with translation proofreading, thereby inhibiting protein synthesis (10). Ribosomal protein S12, encoded by *rpsL*, stabilizes the highly conserved pseudoknot structure formed by 16S rRNA; consequently, amino acid substitutions in RpsL affect the higher-order structure of 16S rRNA and confer streptomycin resistance (10).

**FIG 1:**
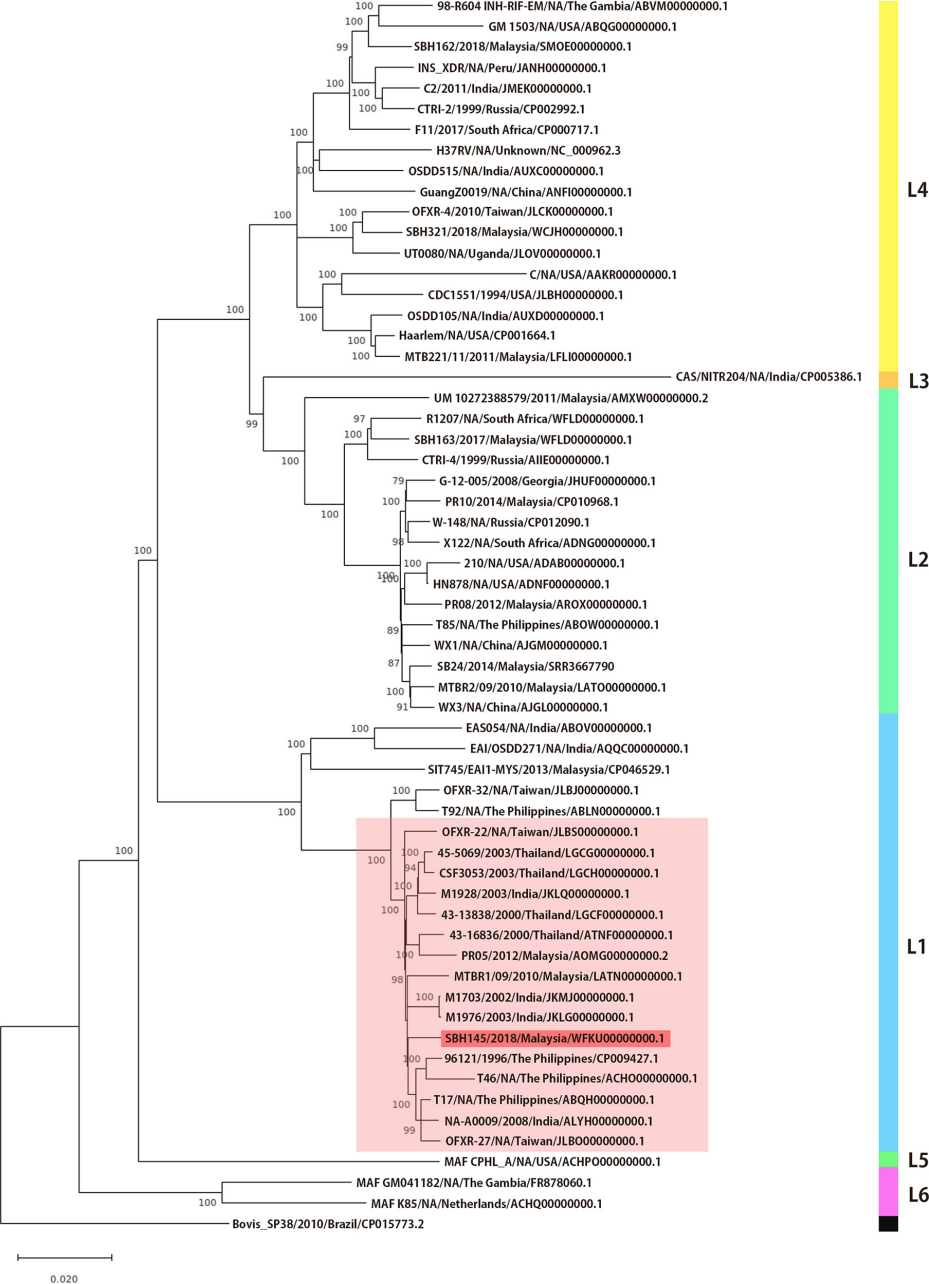
Phylogenetic tree showing that theSBH145 strain belongs to lineage 1 of the EAI2-Manila family and is clustered with strains from Thailand, India, Malaysia, Taiwan, and the Philippines. The phylogenetic tree was constructed using SNP data for the genome sequences of 59 M. tuberculosis strains extracted from GenBank; strains are indicated as strain name/country/accession number. Mycobacterium bovis strain SP38 was used as an outgroup. The numbers adjacent to nodes represent the bootstrap values; values less than 70% are not shown. The significance of branching was assessed by bootstrap analysis of 1,000 replicates. The scale bar shows the genetic distance, which is expressed as nucleotide substitutions per site.

For SNP-based phylogenetic analysis, core SNPs were generated by the kSNP3 package (11), nucleotide sequences were aligned, and the maximum likelihood method with the general time-reversible model was used (12). Strain SBH145 clustered with strains from peninsular Malaysia, the Philippines, Thailand, India, and Taiwan (Fig. 1), which are not streptomycin resistant, and it belongs to the Manila clade of lineage 1 of the M. tuberculosis complex (13–15). Default parameters were used for all software except where otherwise noted.

### Data availability.

Raw reads have been deposited in the NCBI SRA under accession number SRR10204507, with BioSample accession number SAMN12878104 and BioProject accession number PRJNA575111. This whole-genome shotgun project has been deposited in DDBJ/ENA/GenBank under accession number WFKU00000000.1.
